# Nursing Students' Competency about Writing Nursing Care Plan: An Exploratory Study in Iran

**DOI:** 10.1155/2024/6653850

**Published:** 2024-01-12

**Authors:** Ali Jabraeelzadeh Kamblash, Mohammad Javad Jafari, Reza Nemati-Vakilabad, Mohammad Reza Mojebi, Pouya Mostafazadeh, Alireza Mirzaei

**Affiliations:** ^1^Students Research Committee, School of Nursing and Midwifery, Ardabil University of Medical Sciences, Ardabil, Iran; ^2^Student Research Committee, School of Nursing and Midwifery, Guilan University of Medical Sciences, Rasht, Iran; ^3^Department of Emergency Nursing, School of Nursing and Midwifery, Ardabil University of Medical Sciences, Ardabil, Iran

## Abstract

**Background:**

By utilizing the nursing care process, healthcare professionals can implement nursing knowledge effectively and efficiently, ultimately leading to a significant improvement in the quality of care provided.

**Objective:**

This study aimed to assess writing nursing care plan competence among Ardabil nursing students in northwestern Iran.

**Methods:**

The study involved 248 nursing students from the second to eighth semesters in three nursing and midwifery schools located in the Ardabil province of northern Iran. The data were collected using a simple random sampling method and included a demographic questionnaire along with a survey questionnaire on nursing care plan writing skills. This was conducted between May and June 2023. Descriptive statistics, Pearson correlation, *t*-test, ANOVA, and multiple linear regression analyses were conducted for data analysis.

**Results:**

The mean score (SD) for writing a nursing care plan in a student survey was 3.35 (0.57) on a scale of 1 to 5. The survey's weighted mean score for each dimension is as follows: data gathering (*D*) = 3.40 (0.73), identification of client's problems (*P*) = 3.40 (0.73), sustainable goals (*G*) = 3.31 (0.77), appropriateness of intervention (*I*) = 3.30 (0.67), and recognizing outcomes (*O*) = 3.37 (0.69). This indicates that the students performed well overall. However, the lowest score was in the appropriateness of interventions dimension. Multiple linear regression analysis revealed that four variables, mean score of grade point average (GPA), age, academic term, and time spent studying (hours per day), were significant predictors of the student survey's scores on writing nursing care plans. These variables could predict 61% of the total variance. *Implication for Nursing Management*. Nursing school administrators can establish programs to improve education and practice in nursing care plan development based on this study. They can use these findings to enhance nursing education and practice for students, which will ultimately lead to better nursing care plans. Administrators should ensure that students have the necessary skills for writing efficient care plans and provide training courses to improve their writing and critical thinking skills. Policies can be implemented to encourage students to increase their study hours and maintain quality care programs.

**Conclusion:**

Nursing students are skilled in creating well-written nursing care plans that follow the nursing process. The quality of these plans improves with higher GPAs and longer study hours. To enhance nursing care plans, nursing education should focus on developing skills and allocating sufficient time for studying and practicing. Collaborating with faculty and administrators, as well as using reliable resources, can also help improve the quality of care plans.

## 1. Introduction

Nursing care is a critical aspect of nursing practice that involves efficiently managing care for patients [1]. The nursing process is a scientific approach that is widely accepted and involves five stages: assessment, nursing diagnosis, planning, implementation, and evaluation [2, 3]. The nursing process is a problem-solving method that uses scientific reasoning, critical thinking, and problem-solving skills to guide nurses in providing individualized patient care according to their unique needs [1, 2]. The primary objective of nursing care is to provide high-quality care that enhances patient outcomes [4, 5]. The nursing process is a fundamental policy and principle for nursing care and it promotes analytical reasoning and standardizes performance among nurses [3]. Its effective implementation in patient care enhances nursing efficiency, facilitates care documentation, and provides a unified language for the nursing profession [6]. Nursing is a globally accepted approach in clinical practice to deliver quality, individualized patient care [2]. Nursing students are expected to demonstrate high competency levels in clinical settings [7].

Healthcare providers must possess practical knowledge, skills, and attitudes to carry out care tasks effectively [8]. Maintaining high standards of nursing performance is a challenge for the healthcare sector of any country [9]. Weak nursing competencies can result in low-quality healthcare and increased mortality rates [2]. According to a study by Seçer and Karaca, 93.4% of nurses believed the nursing process should be used for care [1]. Another survey by Zeleke et al. reported that 74.7% of nurses followed the process [3]. Assessing the skills of nursing students can provide accurate information for better planning and management at educational and practical levels [10]. Precise educational strategies are necessary to improve future nursing competencies [11].

Nursing education management is crucial for bridging the gap between theory and practice in the nursing process. Assessing the skills and effectiveness of nursing students is essential for their development and improvement in the nursing process [10]. Evaluating the quality of nursing process development by students helps enhance their competencies in patient care management [5]. Nursing instructors need a clear understanding of critical competencies in nursing to effectively support their students and seize opportunities to assist them [11]. Nursing students must be well prepared and qualified to plan care and emphasize accurate documentation for patient safety. Mastering the art and science of writing a nursing process is crucial for every student [5]. At the undergraduate level, nursing students require varying support, supervision, and regulatory guidance to learn skills such as physical examination and nursing processes while gaining practical experience [12].

Implementing the nursing process in hospitals is challenging due to factors such as lack of time, inadequate training, insufficient managerial support, low motivation, shortage of specialized individuals, unclear job descriptions, and low salaries [2, 6]. One solution is to involve nursing mentors who can provide courses and active learning strategies to improve nursing students' competency. National policy frameworks and interventions are necessary to enhance education and the implementation of the nursing process in clinical settings [2, 3, 6]. Adequate staffing levels and reduced workloads for individual nurses are crucial for effectively using the nursing process and improving patient care quality [6]. Standardizing nursing process documents across all hospitals can streamline the process and enhance problem-solving skills [3]. Positive attitudes and nursing students' ability to develop and implement the nursing process can improve the quality of patient care [2, 13–15].

Optimizing the nursing process is essential for providing quality patient care, reducing expenses, and minimizing medical errors. Focusing on research in this area can lead to better patient outcomes, lower healthcare costs, and improved community health. Nursing students have a unique opportunity to develop their nursing process development skills, contribute to enhancing healthcare outcomes, and improve the quality of patient care. This study aims to evaluate the competency of Ardabil nursing students in writing nursing care plans in Northwestern Iran.

## 2. Materials and Methods

### 2.1. Setting, Sample, and Data Collection

A cross-sectional, descriptive study was conducted to investigate the competencies of nursing students in writing nursing care plans. The study included nursing students from the second to eighth semesters, studying at nursing schools in Ardabil province, located in northern Iran. The Ardabil province has three nursing and midwifery schools: Ardabil, Meshgin, and Germi. Nursing students in Iran learn the nursing process in theoretical courses such as principles and skills of nursing and nursing concepts in the first semester, which they use during their internships. First-semester students were excluded from the study as they had yet to pass the nursing process unit. The total number of nursing students in these schools was 667. Using Epi Info StatCalc version 7 software, a sample size of 244 was estimated at a 95% confidence level and a 0.05 margin of error. Considering a nonresponse rate of 10%, the final sample size was determined to be 268 individuals. The sample attrition rate in this study was 8%, with 22 incomplete questionnaires removed due to missing data. The required number of samples from each center was distributed among second-, third- and fourth-year students based on their population size. Therefore, students from each year were randomly selected for the study (152 from Ardabil nursing and midwifery school, 51 from Germi nursing school, and 45 from Meshgin nursing school). Data from 248 samples were analyzed and collected face-to-face based on the student's educational program from May to June 2023.

### 2.2. Measures

Data were collected through a two-part questionnaire: (1) sociodemographic variables and (2) student survey on writing a nursing care plan.

#### 2.2.1. Sociodemographic Variables

The data collection tools comprised a demographic information questionnaire and a student survey on writing a nursing care plan. The demographic information questionnaire included questions related to demographic characteristics such as age, gender, marital status, academic term status, grade point average (GPA)—which is graded on a scale of 0 to 20, interest in nursing commitment, nursing school or city of study, participation in a nursing process course (yes or no), interest in the nursing field (yes or no), and time spent studying (in hours per day).

#### 2.2.2. The Student Survey on Writing Nursing Care Plan Questionnaire

In 2020, Salvador et al. developed a questionnaire to evaluate the competency of nursing students in developing nursing care plans. The student survey on writing a nursing care plan consists of 25 items categorized into five domains: (1) data gathering (*D*); (2) identification of client's problems (*P*); (3) sustainable goals (*G*); (4) appropriateness of intervention (*I*); and (5) Recognizing outcomes (*O*). Participants respond to questions using a 5-point Likert scale (poor = 1, fair = 2, good = 3, very good = 4, excellent = 5), and the overall mean score is interpreted from poor to excellent [5]. This questionnaire was translated and cross-culturally adapted in Iran by Ramezanzadeh et al. [10] in 2023. Permission to use the original questionnaire [5] and the translated version in Iran was obtained. Internal consistency was assessed using Cronbach's alpha coefficient, which was 0.93 in Salvador's study and 0.95 in Ramezanzadeh et al.'s study [10]. Our analysis yielded a Cronbach's alpha coefficient of 0.91, indicating high internal consistency among the measured variables.

### 2.3. Ethical Considerations

The Ethics Committee of Ardabil University of Medical Sciences approved the present study with a corresponding ethics code (code number = IR.ARUMS.REC.1402.034). Eligible individuals interested in participating were informed about the study's objectives and encouraged to participate voluntarily. Only those who provided written informed consent and willingly agreed to participate were given the questionnaire. The confidentiality of their responses was ensured, and there would be no adverse consequences resulting from their refusal to participate. All researchers involved in the study were responsible for distributing and collecting the questionnaires, and they took full responsibility for ensuring that the participant's privacy and confidentiality were maintained. The questionnaire was self-reported, and it took 5 to 15 minutes to complete. The first page of the questionnaire included details of the study objectives, confidentiality, anonymity, and privacy of the respondents. The participants' responses were kept confidential, and the researchers involved were responsible for distributing and collecting the questionnaires.

### 2.4. Data Analysis

To describe the demographic characteristics and research variables, descriptive statistics such as percentages, frequencies, mean, and standard deviation were used. The questionnaire items were analyzed using mean, standard deviation, and a 95% confidence interval. *T* test, ANOVA, and Pearson correlation were used to examine the difference and relationship between the main study variable and demographic factors of nursing students. To evaluate the predictors of student surveys on writing nursing care plans, multiple linear regression analysis was employed, using demographic factors as independent variables. A significance level of 0.05 was set for this study.

## 3. Results

A total of 248 nursing students participated in this study, comprising 134 females (54%) and 114 males (46%). They had a mean GPA of 16.37 (1.29) on a scale of 0–20. Out of the total participants, 231 (85.9%) were single. The study included 152 students (61.3%) from the midwifery nursing department in Ardabil. Among them, 207 (83.5%) had not completed the nursing process in their education, while 199 (80.2%) showed a keen interest in nursing. In addition, 96 students (38.7%) studied for one to two hours daily. You can check Table 1 for more information on the demographic characteristics of the participants.

The scores of Table 2 from the survey questionnaire for student survey on writing nursing care plans were organized into domains. In the data gathering subgroup, the item that received the highest rank mean score 3.45 (standard deviation (SD) = 0.98) was “I utilize a systematic way in collecting my objective data like Gordon's Assessment or any other pertinent assessment tool,” while the lowest level among nursing students 3.33 (0.83) was for the item “I utilize therapeutic communication throughout patient assessment.” In the identifying clients' problem subgroup, the highest-ranked phrase 3.43 (0.95) was “I am aware of the various ways on how to formulate good nursing diagnoses,” while the lowest-ranked item 3.34 (1.02) was “I use and refer to NANDA resources every time I formulate my client's nursing diagnosis.” In addition, in the formulating sustainable goals subgroup, the item that obtained the highest rank 3.34 (1.07) was “I specify in my planning the recipient and reason for formulating the nursing care plan,” while the lowest-ranked item 3.28 (0.99) was “I put a time frame whenever I formulate my nursing care plan for evaluation.” In the providing appropriate interventions subgroup, the item that received the highest score 3.35 (1.01) was “I base my independent interventions on the identified needs of my patient,” while the lowest score 3.20 (0.96) was received by the item “I incorporate interdependent collaborations with various healthcare departments whenever I plan care for my client.” Finally, in the recognizing Client's outcomes subgroup, the item that received the highest score 3.45 (0.95) was “I seek back to my planning's goals and objectives if I have achieved my SMART plan.” while the lowest score 3.29 (1.09) was received by the item “I review my nursing care plan and decide whether to terminate, continue and change it after a series of evaluation.” The overall mean score of the student survey on writing nursing care plans was 3.35 (0.57) on a scale of 1 to 5.

Table 2 presents the weighted mean score and interpretation for each dimension. The scores we obtained are as follows: (a) data gathering (*D*) = 3.40 (0.73), which is considered good; (b) identification of client's problems (*P*) = 3.40 (0.73), which is also good; (c) sustainable goals (*G*) = 3.31 (0.77), again, good; (d) appropriateness of intervention (*I*) = 3.30 (0.67), good; and (e) recognizing outcomes (*O*) = 3.37 (0.69), which is, again, good. The overall weighted mean score is 3.35 (0.57), indicating that the students' nursing care plan (NCP) writing skills are of good quality. The dimension with the lowest score was the appropriateness of intervention.

Table 3 summarizes the relationship between the student survey on writing a nursing care plan and the demographic factors of nursing students. It shows the study's overall mean scores and range based on the participants' demographic characteristics. The results indicate a significant positive correlation between the overall mean score of the student survey on writing a nursing care plan and the mean score of grade point average (GPA), academic term, interest in nursing commitment, interest in the nursing field, and time spent studying. On the other hand, there is a significant inverse correlation between the overall mean score of the student survey on writing a nursing care plan and age.

The results of multiple regression analysis, indicating the predictors of the student survey on writing a nursing care plan, are shown in Table 4. A considerable variable regression analysis was conducted using the student survey on writing a nursing care plan as the dependent variable and demographic characteristics as the independent variable. Of these ten variables, 4 were significant predictors of the student survey on writing a nursing care plan. The regression model's coefficient of determination (*R*^2^) indicates that 61% of the total score of the student survey on writing a nursing care plan can be explained by the input variables in the model. Among the input variables in the model using the ENTER method, as reported in the table, there was a statistically significant relationship between mean GPA, age, academic term, and time spent studying (hours per day).

## 4. Discussion

Nursing students play a crucial role in providing continuous care and improving the healthcare system in the future. The nursing process serves as a framework for nursing care, an essential tool for implementing nursing knowledge that enhances the quality of care. Writing nursing care plans is a skill and competency identified in the nursing profession. This study was conducted to investigate nursing students' competence in writing nursing care plans.

Although students showed a good score in writing NCP, previous studies have indicated that nursing students require appropriate training to improve their implementation of the nursing process [6, 16]. There are several barriers to implementing the nursing process, such as high patient-to-nurse ratios, inadequate and improper training, lack of continuous supervision and control, insufficient time allocated for training, and lack of retraining opportunities [17]. Nursing students must be familiar with real-life conditions and appropriate tools to effectively implement the process. Addressing these obstacles can lead to better outcomes. Improper implementation of the nursing process is often due to insufficient education, inadequate attitudes, and a lack of training in nursing schools [18]. Nurses who are more familiar with the nursing process and use it in their care have a more positive attitude towards it [3]. The nursing process enhances the quality of nursing care by ensuring individualized and high-quality care [6, 19]. To ensure mastery and translation into practice, nursing educators should use active teaching strategies [6]. Patient care skills and the nursing process should be assessed and monitored through evaluation courses [17].

The results showed nursing students have good writing skills for creating nursing care plans (NCPs). They are proficient in data collection during the evaluation phase, with an average score of 3.40. In a study by Salvador et al., nursing students performed excellently [5]. Assessing patients is the first step in nursing and guiding interventions [20]. Nurses' initial patient assessment translates theory into practice and demonstrates the value of knowledge, skills, and attitudes in providing individualized care to complex healthcare needs. It is crucial for nursing diagnosis and the nursing process [3]. In Namibia, nursing students may miss out on physical examination aspects due to not completing all stages of nursing. This can lead to incorrect diagnoses as each stage of nursing is interrelated and a failure in one stage can affect other stages [21]. The difference in mean data collection between the two communities is due to cultural differences [5].

Among the data collection items, the lowest score was related to “I use therapeutic communications throughout the patient assessment.” Good communication and collaboration are important competencies in patient-centered care and nursing practice. Students' perception highlights that nursing is influenced by various aspects of patient care, including patient preferences and effective communication. Language barriers and patients' preferences for professional nurses may hinder nursing students' ability to apply the nursing process [11, 21].

Based on our results, in the second component of the survey, students had a good quality level in writing NCP, meaning that nursing students have good foundations during problem identification or nursing diagnoses (*P*=3.31). As per Salvador et al., the lowest score was related to “I use NANDA nursing diagnosis resources every time I formulate a nursing diagnosis.” Nursing diagnosis should be based on assessment results and used as a reference for interventions [22]. Nurses and nursing students should apply the nursing process to enhance patient care quality and safety [23]. Nursing students learn education topics and use the nursing process to understand patient health problems and study evidence [24]. A nursing diagnosis reflects the student's ability to identify patient problems during the initial assessment. A correct diagnosis leads to proper intervention and evaluation, while an incorrect diagnosis can have the opposite effect. Identifying patient problems is critical for quality care [25].

The study results indicated that in the third dimension, nursing students also had good quality in developing nursing processes, meaning they had reasonable primary goals (*G*) or planning for patient nursing diagnoses. In Salvador et al. study, most nursing students felt the management course provided enough knowledge to set patient goals. Nursing mentors should teach SMART goal-setting principles [5]. A review in Iran found that the nursing process is not commonly used in low-income countries like Iran, Ethiopia, Kenya, and Taiwan. Reasons include a lack of awareness among nursing faculty about the process, inadequate hospital infrastructure, and insufficient support from nursing organizations [26]. It is imperative to adopt and implement appropriate policies that ensure nursing students can confidently execute the nursing process in the clinical environments of low-income countries on a widespread and continuous basis.

The lowest score among the items was related to “I set a time frame when scheduling my nursing care plan for evaluation.” The nursing process is challenging, time-consuming, and difficult to perform. Taking care of multiple patients simultaneously can disrupt decision-making. Software can help manage time and streamline the process [26]. Nurses cite workload and staff shortages as obstacles to using the nursing process effectively in patient care, as per Bassah et al. and colleagues [6]. It is essential that managers take prompt action to overcome any obstacles hindering nurses from implementing the nursing process effectively. By doing so, nurses will have adequate time to provide individualized care to patients, leading to better health outcomes.

According to the study results, nursing students had good-quality writing NCP in the fourth dimension. Nursing students could use appropriate interventions (*I*) based on sound planning. In the Salvador et al.'s study, students achieved a perfect score. In the Sendir et al.'s study, nursing students faced difficulty determining nursing interventions [27]. The appropriate diagnosis of intervention depends on the nurse's knowledge and awareness of the disease and the patient's status. According to Zeleke et al., nurse awareness is the most predictive factor for the nursing process [3]. Students had the lowest score in the fourth-dimension item, “Whenever I intend to take care of my patient, I consider the collaborative efforts with various healthcare departments.” One of the issues surrounding nursing profession development includes physician dominance in healthcare systems. When nurses demonstrate sufficient ability to improve the quality of care they provide, other health teams can identify the nursing profession [11].

The study results indicated students exhibited high-quality nursing writing skills in the fifth dimension. This finding meant that nursing students had good foundations in evaluating outcomes (*O*) or evaluating interventions performed. However, in the Salvador et al.'s study, students had achieved excellent grades. Nursing students must master the art of writing a care plan using all available resources to ensure optimal patient health [5]. Among the items related to the fifth dimension, the lowest score was related to the item “I review my nursing process and decide whether to terminate it after a series of evaluations, continue it, or make changes.” Through the nursing process, nursing students and nurses play a significant role in evaluating nursing care results [9]. The evaluation stage assesses nursing care programs for efficiency, completeness, and adequacy while identifying deficiencies in previous programs [28]. Nursing education is crucial in preparing students with the knowledge and skills needed for effective nursing practice [10]. Nursing instructors shape the future of nursing. They must equip students with the skills to deliver quality care. The nursing process and final evaluation are critical components of patient care. Prioritizing them empowers nurses to deliver the best care and improve patient health outcomes. Let us support the full implementation of the nursing process and final evaluation.

The findings suggest that as students' GPAs increase, their scores in writing nursing care plans rise, which is consistent with prior research [29]. According to a study by Vasli et al., GPA is a significant predictor of clinical competency scores among nursing students [29]. It is possible that academic skills have an impact on clinical skills, which could explain the results. Students who excel in their academic studies and have high GPAs may have a better understanding of nursing concepts, which can have a positive impact on their clinical skills. On the other hand, students with lower GPAs may need to have a deeper understanding and application of the nursing process. Therefore, it is crucial for students who did not receive satisfactory GPAs during their academic years to enhance their competence in writing nursing process formulations through more practice and study. However, further research is required to comprehend and confirm this relationship fully.

According to the results of the present study, as students' progress through their academic terms, their competency scores in nursing process formulation increase, which is consistent with the findings of Huang et al. According to Huang et al. study, first- and second-year nursing students had lower nursing competency scores than third- and fourth-year students [11]. The use of the nursing process depends on many factors, including knowledge and skills that ideally should be developed in nursing students throughout their education [21]. In addition, as students' age, their competency scores in nursing process formulation also increase. This finding contradicts the results of Bassah et al.'s study [6]. These findings may be due to improved clinical experience and practice in formulating the nursing process with age and longer academic terms.

The findings of this study suggest that an increase in nursing students' time spent studying (hours per day) can lead to an improvement in their ability to develop nursing processes. This may be due to the higher level of knowledge that nursing students possess due to increased studying and staying up-to-date with the latest information. Therefore, nursing instructors and managers should promote programs that enhance students' academic motivation [30]. Nursing is a dynamic and demanding profession requiring high knowledge and experience [31]. As such, nursing students should continuously study to become familiar with the latest advancements in the field and provide the best possible care to patients. In addition, increased studying helps students perform better in nursing and cope with this profession's complex and risky conditions.

### 4.1. Limitations

The study's small sample size of nursing students presents a significant limitation, which may compromise the generalizability of the results. To ensure more accurate and reliable outcomes, it is imperative for future studies to consider evaluating nursing care programs with larger sample sizes. It is important to note that this study solely focused on undergraduate nursing students at Ardebil University of Medical Sciences; thus, caution should be exercised when applying these findings to the entire population of Iran. In order to enhance the generalizability of the findings, it is highly recommended to conduct multicenter studies involving students from various universities in Iran as well as different regions of the world. Lastly, it is crucial to address the limitations of this study by conducting further research in the future, which will contribute to a more comprehensive understanding of the topic.

### 4.2. Implications for Nursing Management

Nursing schools must prioritize the development of practical care plan writing skills among their students. Teachers should use effective teaching methods and implement comprehensive training programs to facilitate learning [32]. Students should also be encouraged to aim for greater efficiency and create high-quality care programs. These initiatives will help to advance research methodologies and improve nursing studies. Investing in these efforts will ensure that nursing education meets the needs of society and empower students to promote community health.

## 5. Conclusions

According to the study, nursing students can create high-quality writing nursing care plans. Nurses are essential as caregivers, coordinators, educators, and information sources [33]. The study found that nursing students with higher GPAs and more time studying produced better nursing care plans. Therefore, it is recommended that students focus on improving their skills in various courses during their training period. They should strive to have a deep and accurate understanding of nursing concepts and principles and devote sufficient time to studying and practicing nursing care plans. Using reliable and up-to-date resources in nursing care planning and collaborating with faculty members and school administrators can also help improve the quality of nursing care programs. Based on these findings, school nursing administrators can develop programs to enhance the quality of education and training in nursing care planning for students. However, further studies are needed to conduct additional qualitative research to analyze the ability of nursing students to write care plans.

## Figures and Tables

**Table 1:** Sociodemographic characteristics of the study sample (*N* = 248).

Characteristics	Categories	Mean (*n*)	SD (%)
Grade point average (grading is based on a 0 to 20 score)		16.37	1.296

Age	18–22	122	49.2
	23–27	126	50.8

Gender	Female	134	54.0
	Male	114	46.0

Marital status	Single	213	85.9
	Married	35	14.1

Semester	2	23	9.3
	3	47	19.0
	4	23	9.3
	5	56	22.6
	6	26	10.5
	7	55	22.2
	8	18	7.3

Interest for nursing commitment	*Yes*	54	21.8
	*No*	194	78.2

Nursing school	Ardabil	152	61.3
	Meshgin	45	18.1
	Germi	51	20.6

Nursing process course	Yes	41	16.5
	No	207	83.5

Interest in the nursing field	Yes	199	80.2
	No	49	19.8

Time spent studying (hours per day)	Less than 1 hour	71	28.6
	Between 1 and 2 hours	96	38.7
	Between 2 and 3 hours	72	29.0
	More than 3 hours	9	3.6

**Table 2:** The status of Iranian nursing student survey on writing nursing care plan (*N* = 248).

Items	Mean	SD
*Data gathering (D)*	3.40 good	0.734
(1) I utilize therapeutic communication all throughout the duration of patient assessment	3.33	0.831
(2) I maximize my time during the entire course of patient-nurse interaction	3.40	0.985
(3) I find it easy to recognize irregularities from normal to abnormal changes based on my assessment	3.38	0.915
(4) I utilize systematic way in collecting my objective data like Gordon's Assessment or any other pertinent assessment tool	3.45	0.980
(5) I make sure that the client's given data are coherent	3.42	0.923

*Identification of client's problems (P)*	3.40 good	0.738
(6) I determine my client's needs based from my assessment based from Maslow's hierarchy of needs	3.42	0.999
(7) I categorize my client's needs based from the various types of nursing diagnoses like actual, risk, wellness, syndrome, and possible	3.42	0.991
(8) I am aware of the various ways on how to formulate good nursing diagnoses	3.43	0.958
(9) I understand the different terminologies used in formulating nursing diagnosis	3.35	1.039
(10) I use and refer to NANDA resources every time I formulate my client's nursing diagnosis	3.34	1.034

*Sustainable goals (G)*	3.31 good	0.777
(11) I specify in my planning the recipient and reason for formulating the nursing care plan	3.34	1.078
(12) I look on the measurable quantifiers that will be used as parameters for my planning	3.33	1.052
(13) I make sure that the goals and objectives I set for my nursing care plan are attainable	3.30	1.038
(14) I see to it that my nursing care plan is realistic and does not only base from imagination	3.31	1.062
(15) I put time frame whenever I formulate my nursing care plan for evaluation	3.28	0.998

*Appropriateness of intervention (I)*	3.30 good	0.679
(16) I assessed my patient before I start my interventions to come up with baseline data and use it as my parameters for ongoing evaluation	3.32	0.978
(17) I based my independent interventions based from the identified needs of my patient	3.35	1.012
(18) I put stand-alone rationales in all the nursing interventions I plan to implement for my patient	3.28	0.965
(19) I resort getting doctor's orders when I am planning for my nursing interventions especially with pharmacological, medical and surgical treatments and modalities	3.32	0.990
(20) I incorporate interdependent collaborations with various healthcare departments whenever I plan care for my client	3.20	0.966

*Recognizing outcomes (O)*	3.37 good	0.690
(21) I re-assess my patient after all the nursing interventions done	3.38	1.007
(22) I go back to my nursing diagnosis if my nursing care plan answered the client's health needs	3.40	0.989
(23) I seek back to my planning's goals and objectives if I have achieved my SMART plan	3.45	0.955
(24) I evaluate all the nursing interventions done to my client to check and validate its appropriateness and effectiveness	3.35	1.062
(25) I review my nursing care plan and decide whether to terminate, continue and change it after series of evaluation	3.29	1.093

*Total*	3.35 good	0.576

*Note*. Interpretation: excellent = 4.21–5.0; very good = 3.41–4.20; good = 2.61–3.40; fair = 1.81–2.60; poor = 1.00–1.80.

**Table 3:** Relationship between nurses' sociodemographic characteristics and student survey on writing nursing care plan (*N* = 248).

Characteristics	Categories	Mean	SD	Statistical	*p* value
Grade point average		16.37	1.296	*r* = 0.688	*p* < 0.001

Age	18–22	3.04	0.468	*t* = −9.816	*p* < 0.001
	23–27	3.65	0.509		

Gender	Female	3.32	0.556	*t* = −0.907	*p*=0.365
	Male	3.39	0.599		

Marital status	Single	3.34	0.580	*t* = −0.951	*p*=0.343
	Married	3.44	0.551		

Semester	2	2.88	0.529	*F* = 34.77	*p* < 0.001
	3	3.00	0.458		
	4	3.08	0.347		
	5	3.22	0.477		
	6	3.35	0.328		
	7	3.99	0.330		
	8	3.72	0.508		

Interest for nursing commitment	*Yes*	3.54	0.518	*t* = 2.733	*p*=0.007
	*No*	3.30	0.582		

Nursing school	Ardabil	3.33	0.550	*F* = 1.467	*p*=0.233
	Meshgin	3.31	0.583		
	Germi	3.48	0.638		

Nursing process course	Yes	3.34	0.480	*t* = −0.162	*p*=0.872
	No	3.36	0.594		

Interest in the nursing field	Yes	3.42	0.557	*t* = 3.99	*p* < 0.001
	No	3.07	0.569		

Time spent studying (hours per day)	Less than 1 hour	3.01	0.462	*F* = 34.58	*p* < 0.001
	Between 1 and 2 hours	3.26	0.501		
	Between 2 and 3 hours	3.71	0.510		
	More than 3 hours	4.16	0.143		

**Table 4:** Multiple regression analysis predicting student survey on writing nursing care plan according to sociodemographic characteristics (*N* = 248).

Variables	*B*	S. E	Beta	*t*	Sig	95.0% confidence interval for *B*	
						Lower bound	Upper bound
(Constant)	−1.247	0.402		−3.102	0.002	−2.039	−0.455
Age	0.164	0.071	0.142	2.323	0.021	0.025	0.303
Gender	0.050	0.049	0.043	1.028	0.305	−0.046	0.145
Marital status	0.050	0.071	0.030	0.708	0.479	−0.090	0.190
Semester	0.071	0.023	0.226	3.047	0.003	0.025	0.118
Grade point average	0.216	0.022	0.486	10.044	0.000	0.174	0.258
Interest for nursing commitment	−0.028	0.066	−0.020	−0.423	0.673	−0.157	0.102
Nursing school	0.002	0.033	0.003	0.059	0.953	−0.063	0.067
Nursing process course	0.083	0.066	0.053	1.248	0.213	−0.048	0.214
Interest in the nursing field	0.052	0.067	0.036	0.784	0.434	−0.079	0.184
Time spent studying (hours per day)	0.079	0.038	0.116	2.058	0.041	0.003	0.155

*R* ^2^ = 0.61, adjusted *R*^2^ = 0.59, *F* (37.43), and *p* < 0.001.

## Data Availability

The data supporting the results of this study are available on request from the corresponding author. The data are not publicly available due to privacy or ethical restrictions.
